# Circulating nitric oxide pathway metabolites in heart failure with preserved ejection fraction: a sex-stratified cross-sectional analysis

**DOI:** 10.1186/s13293-026-00940-7

**Published:** 2026-06-15

**Authors:** Sophia Marie-Theres Dinges, Edzard Schwedhelm, Flavia Baldassarri, Bernhard Haller, Andreas B. Gevaert, Rainer Böger, Ephraim B. Winzer, Frank Edelmann, Ulrik Wisløff, Volker Adams, Burkert Pieske, Emeline M. Van Craenenbroeck, Martin Halle, Susanna M. Hofmann, Stephan Mueller-Stegner

**Affiliations:** 1 TUM School of Medicine and Health, Department for Preventive Sports Medicine and Sports Cardiology, Technical University of Munich, TUM University Hospital, Munich, Germany; 2 DZHK (German Centre for Cardiovascular Research), Partner Site Munich Heart Alliance, Munich, Germany; 3 Research Group “Women and Diabetes”, Institute for Diabetes and Regeneration, Helmholtz Center Munich - German Research Center for Environmental Health, Neuherberg, Germany; 4 Institute of Clinical Pharmacology and Toxicology, University Medical Center Hamburg- Eppendorf, Hamburg, Germany; 5 DZHK (German Centre for Cardiovascular Research), Partner Site North, Hamburg, Germany; 6 TUM School of Medicine and Health, Institute of AI and Informatics in Medicine, Technical University of Munich, Munich, Germany; 7 Department of Cardiology, Antwerp University Hospital, Edegem, Belgium; 8 Research Group Cardiovascular Diseases, GENCOR Department, University of Antwerp, Antwerp, Belgium; 9 Department of Internal Medicine/Cardiology, Heart Center, University Clinic, Technische Universität Dresden, Dresden, Germany; 10 Department of Cardiology, Angiology and Intensive Care Medicine, Deutsches Herzzentrum der Charité (DHZC) - Medical Heart Centre of Charité and German Heart Institute Berlin, Berlin, Germany; 11 Charité - Universitätsmedizin Berlin, Corporate Member of Freie Universität Berlin, Humboldt Universität zu Berlin, Berlin, Germany; 12 DZHK (German Centre for Cardiovascular Research), Partner Site Berlin, Berlin, Germany; 13 Department of Circulation and Medical Imaging, Faculty of Medicine and Health Sciences, Norwegian University of Science and Technology, Trondheim, Norway; 14 Division of Cardiology, Department of Internal Medicine, University Medicine Rostock, Rostock, Germany; 15 Department of Medicine IV, University Hospital, LMU Munich, Munich, Germany; 16 German Center for Diabetes Research (DZD), Neuherberg, Germany

**Keywords:** HFpEF, nitric oxide, endothelial dysfunction, vascular function, SDMA, ADMA, homoarginine, GABR, biomarker, sex differences

## Abstract

**Background:**

Heart failure with preserved ejection fraction (HFpEF) is more prevalent in women, who often present with earlier onset and greater severity of vascular and endothelial dysfunction. Nitric oxide (NO) is a key contributor to vascular health, and several NO-related metabolites are associated with cardiovascular outcomes. However, it remains unknown whether differences in circulating NO-related metabolite concentrations exist between men and women with HFpEF.

**Methods:**

In this cross-sectional analysis of a multicentre randomized controlled intervention trial (OptimEx-Clin), plasma concentrations of NO-related amino acids (L-arginine, L-homoarginine (hArg), L-ornithine, L-lysine, L-citrulline), asymmetric and symmetric dimethylarginines (ADMA, SDMA), and functional metabolite ratios were assessed in 171 patients with HFpEF. Analyses were stratified by sex and age, and further adjusted for relevant clinical covariates using multivariable regression models. A principal component analysis (PCA) was performed to explore sex-related metabolite patterns.

**Results:**

The comparison of NO-related metabolites in 58 men and 113 women with HFpEF showed significant sex differences for SDMA (β-coefficient [95% CI]; -0.11 [-0.20 to -0.03] µmol/L, *p* = 0.010) and hArg (-0.24 [-0.46 to -0.01] µmol/L, *p* = 0.043) in unadjusted models; both with lower concentrations in women. While the association with hArg was attenuated after multivariable adjustment, higher SDMA concentrations in men were observed in five out of six tested models. No other significant sex differences were observed neither in single metabolites nor in composite metabolite ratios. The PCA of all measured metabolites did not demonstrate a clear separation between women and men in the overall metabolite profile.

**Conclusions:**

In HFpEF, sex-related differences in circulating NO-related metabolites were limited and metabolite-specific, with SDMA showing the most consistent sex-associated difference with higher concentrations in men. The substantial overlap of metabolic profiles between women and men showed no distinct patterns of circulating metabolites linked to endothelial function in patients with HFpEF.

**Registry:**

ClinicalTrials.gov, TRN: NCT02078947.

**Supplementary Information:**

The online version contains supplementary material available at 10.1186/s13293-026-00940-7.

## Highlights

This cross-sectional analysis provides sex-stratified plasma concentrations of metabolites of the L-arginine/nitric oxide (NO) pathway and functional metabolite ratios in patients with heart failure with preserved ejection fraction (HFpEF). These metabolites are of interest, as they are involved in the regulation of vascular NO availability and are frequently used circulating biomarker for endothelial function.

Sex-related differences were limited to individual metabolites, with SDMA being higher in men and showing the most robust sex difference even after adjustment for relevant clinical covariates. Circulating NO-related metabolite profiles showed a substantial overlap between women and men, indicating no distinct metabolite patterns across sexes and suggesting that circulating NO-related plasma metabolites are not markedly sex-differentiated in patients with HFpEF.

**Supplementary Information:**

The online version contains supplementary material available at 10.1186/s13293-026-00940-7.

## Plain summary

Heart failure with preserved ejection fraction (HFpEF) is a common form of heart failure that affects more women than men. Differences between women and men with HFpEF have been described, particularly in relation to blood vessel function, but the underlying biological mechanisms are not fully understood. Nitric oxide (NO) is an important messenger molecule and regulator of blood vessel function. Several small molecules in the blood, e.g. the amino acid L-arginine and related compounds, regulate the production of NO. Some of them have been linked to diseases of the blood vessels and the heart. In this study, we investigated whether these molecules differ between women and men with HFpEF. Therefore, we analysed blood samples from 171 patients with HFpEF and measured a range of NO-related molecules in the blood. We then compared plasma levels of these molecules between women and men while considering other clinical factors that may influence these molecules. We found that most of these molecules showed no meaningful differences between women and men. Only one molecule (SDMA) was consistently higher in men compared to women. When considering all measured molecules together, the overall patterns of these molecules showed a considerable overlap between men and women. These results indicate that sex-related differences in HFpEF may not be strongly reflected in circulating molecules of the NO pathway. On the other hand, concentrations of NO-related molecules may be affected by comorbidities and clinical characteristics.

**Supplementary Information:**

The online version contains supplementary material available at 10.1186/s13293-026-00940-7.

## Introduction

Heart failure with preserved ejection fraction (HFpEF) is more prevalent in women, who are typically older at disease onset than men. Although hospitalisation rates were similar between women and men, a recent meta-analysis reported higher all-cause and cardiovascular (CV) mortality in men [1]. HFpEF is a heterogeneous condition with a high comorbidity burden, resulting in multiple disease phenotypes that may be partly shaped by sex-related biological and cardiometabolic differences. Women with HFpEF more frequently present with cardiometabolic risk factors such as hypertension, obesity, diabetes, and systemic inflammation, whereas men more often present with an ischemic phenotype [2, 3]. These risk factors are closely linked to endothelial dysfunction and vascular alterations, both considered central mechanisms of HFpEF progression. Further, vascular stiffness contributes to impaired ventricular-vascular coupling and exercise intolerance, a cardinal feature of HFpEF, which has been shown to be more pronounced in women than in men, both in pre-HFpEF stages [4] and in established HFpEF [5].

Nitric oxide (NO) is a key regulator of vascular and endothelial homeostasis, and reduced NO bioavailability is associated with endothelial dysfunction (ED) and impaired CV health [6]. Circulating NO-related metabolites, such as L-arginine, L-homoarginine (hArg), and asymmetric and symmetric dimethylarginine (ADMA, SDMA), reflect aspects of endothelial NO metabolism and may provide insight into HFpEF pathophysiology [7]. Several of these metabolites have been associated with prognosis, with low hArg levels and elevated ADMA or SDMA levels being linked to adverse CV outcomes [8, 9]. While some sex- and age-related reference values for these metabolites have been reported in healthy populations [10–12], sex-stratified data in patients with CV disease remain limited, and their utility in HFpEF remains uncertain. Notably, several routinely used thresholds of established biomarkers for diagnosis and risk stratification, such as natriuretic peptides, are not sex-specific, although circulating levels are known to be influenced by sex [13]. Furthermore, clinical guidelines for HFpEF management do not provide sex-specific treatment recommendations, largely due to limited evidence [14]. This underscores the need for more detailed research on sex-related differences in HFpEF.

In a previous analysis, we observed that plasma levels of several NO metabolites in patients with HFpEF appeared to differ depending on clinical characteristics, including comorbidities and sex [15]. However, it remains unclear whether the observed sex-related differences in HFpEF are reflected in systemic NO-related metabolite profiles or whether these biomarkers primarily capture shared downstream pathways of disease or aspects of underlying phenotype burden. A better understanding of these relationships may improve the interpretation of NO-related biomarkers in HFpEF. Therefore, the aim of the present study was to perform a detailed sex-stratified analysis of circulating NO metabolites in a well-characterized HFpEF cohort and to assess whether potential differences in plasma concentrations persist after accounting for relevant clinical characteristics.

## Materials and methods

Data and blood samples presented in this cross-sectional analysis were obtained at the baseline visit of the OptimEx-Clin trial (“Optimizing exercise training in patients with heart failure with preserved ejection fraction”; NCT02078947), a multicentre randomized clinical trial investigating the effect of different exercise modes on exercise capacity (VO_2peak_) in patients with HFpEF [16, 17]. The OptimEx-Clin trial and the secondary analysis of blood samples were approved by the local ethics committee for medical research at the Technical University of Munich (approval ID: 403/13) and by the responsible ethics committees at all participating study sites. All participants provided written informed consent before any study-related assessment was performed.

### Study population

Patients were included between July 2014 and May 2017 in study centres in Belgium (Antwerp) and Germany (Berlin, Leipzig and Munich) when meeting the following inclusion criteria: Age ≥ 40 years, sedentary (structured exercise < 2 × 30 min/week), clinically stable (≥ 6 weeks) symptomatic HFpEF (NYHA class II-III) based on an LVEF ≥ 50% and E/e′ medial ≥ 15 or E/e′ medial ≥ 8 with elevated N-terminal prohormone of brain natriuretic peptide (NT-proBNP) ≥ 220 pg/mL and optimal medical treatment for ≥ 6 weeks. Exclusion criteria were: Non-cardiac causes for HF symptoms, significant valvular or coronary disease, uncontrolled hypertension or arrhythmias, primary cardiomyopathies; significant pulmonary disease (FEV1 < 50% predicted, GOLD III–IV); myocardial infarction in the last 3 months; comorbidity that may influence one-year prognosis; inability to exercise or conditions that may interfere with exercise intervention, or signs of ischaemia during exercise testing [16, 17]. The present analysis included all patients that were analysed within the OptimEx-Clin trial [17] and had available blood samples from the baseline visit. This resulted in a final sample size of 171 patients.

### Clinical data and parameters

Clinical data including demographics, medical history and medication were assessed during the baseline study visit. Further, anthropometric measurements, cardiopulmonary exercise testing [17] and the assessment of vascular and endothelial function [18] were performed as previously described. Predicted values for peak oxygen consumption (%-pred. V̇O_2peak_) were calculated according to reference values of the SHIP study [19]. The estimated glomerular filtration rate (eGFR) was calculated using the 2021 CKD-EPI creatinine equation [20].

### NO metabolites and ratios

Blood samples were taken and processed according to standard operating procedures during the trial and frozen at −80 °C. Patients were instructed to be in a fasting state and took their medication as prescribed before the blood draw. Analyses of NO metabolites L-arginine, hArg, ADMA, SDMA, L-citrulline, L-lysine, and L-ornithine were centrally performed in June 2019 using liquid chromatography coupled with tandem mass spectrometry (LC-MS/MS) technology (Varian 1200 MS, Agilent Technologies, Santa Clara, CA, USA), as previously described [15]. Quality controls (QC) were run in two levels by triplicates. The second analysis was done on the QC samples to assess the coefficient of variation and bias of QC, which was below 15% for all analytes.

Further, the ratios L-arginine/ADMA (endothelial NO synthase substrate/inhibitor), L-arginine/L-ornithine (surrogate measure for arginase activity) and the global arginine bioavailability ratio (GABR; L-arginine/(L-citrulline + L-ornithine)) were calculated for each patient.

### Statistical analysis

All analyses were performed using the statistical software R (version 4.3.2, R Foundation for Statistical Computing). Visualizations were generated using the *ggplot2* package. Clinical characteristics are presented as mean ± standard deviation, median [1st; 3rd quartile] or number (%), as appropriate. Comparisons between men and women were performed using the *compareGroups* package. Continuous variables were analysed using independent t-tests or Mann–Whitney U tests, as appropriate, and categorical variables using χ² or Fisher’s exact tests.

The analysis of the relationship of NO metabolites and sex followed a structured three-step approach: First, to provide a descriptive characterisation, all biomarkers and metabolite ratios are reported as median [1st; 3rd quartile] stratified by sex and presented for each age group. Age-related differences in metabolites and ratios were assessed using linear regression models separately for each sex, and age-sex interactions were additionally evaluated for the overall study population. Model assumptions (normality, homoscedasticity, linearity, and independence of residuals) were evaluated using graphical residual diagnostics and metabolite data was log-transformed if model assumptions were not met.

Second, sex-related differences in individual NO metabolites or ratios were assessed using multiple linear regression models with sex as primary predictor (men vs. women) in all models. A sequential step-wise adjustment strategy was applied to evaluate sex-related associations with NO metabolites beyond the influence of clinically relevant parameters: *Model 1* was unadjusted to estimate the crude association with sex. *Model 2* was adjusted for age, given the age-dependence of NO-related metabolites. *Model 3* additionally included %-predicted VO2peak as a marker of exercise tolerance and HF severity. *Model 4* further adjusted for body mass index (BMI) and high-density lipoprotein (HDL) cholesterol to account for body composition and lipid-related effects on endothelial function. *Model 5* added diabetes, hypertension, coronary artery disease, atrial fibrillation, and eGFR to account for common HFpEF comorbidities and renal function. Finally, *Model 6* additionally included ACE inhibitors, angiotensin receptor blockers, diuretics, and statins because these medications may influence vascular function and renal function.

Results are presented as regression coefficients (β) with 95% confidence intervals (CI) and p-values for two-sided tests. Missing covariate data (*Models 4–6*) were handled using multiple imputation by chained equations (20 imputations, predictive mean matching) using the *mice* package. The number of imputed values for each variable can be found in Suppl. Table 1. Linear regression models were fitted in each imputed dataset and pooled using Rubin’s rules.

Third, to explore potential clustering patterns by sex among NO-related metabolites, a principal component analysis (PCA) was performed using the *prcomp* function and visualized with the *factoextra* package. Data was centred and standardized and the first two principal components (PC1 and PC2) were visualized in a biplot, including 95% confidence ellipses for each sex. Metabolites were displayed as loading vectors to illustrate their relative contribution and orientation within the PCs.

As the analyses involved multiple metabolites and ratios, the results should be considered exploratory, as no adjustment for multiple testing was applied.

## Results

### Study sample

Clinical characteristics of the 171 patients with HFpEF are presented in Table 1. The study cohort comprised 58 men and 113 women. The overall mean age was 69.7 ± 8.4 years with men being significantly older than women (72.2 ± 6.9 vs. 68.4 ± 8.8 years, *p* = 0.003). More men had diabetes, coronary heart disease, and history of myocardial infarction (all *p* < 0.05). Women had significantly higher total, LDL and HDL cholesterol levels (all *p* < 0.001), but were less likely to receive statin therapy than men (44.2 vs. 72.4%, *p* = 0.001). VO_2peak_ was not significantly different between men and women (19.4 ± 5.5 vs. 18.5 ± 5.2 ml/min/kg, *p* = 0.300), however, the %-predicted VO_2peak_ was higher in women than in men (93.5 ± 21.7 vs. 85.5 ± 21.1%, *p* = 0.022). Among those with available data on vascular function, women had higher reactive hyperaemia index (RHI) (2.32 ± 0.74 vs. 1.99 ± 0.60, *p* = 0.014) and augmentation index (Aix) (35.9 ± 8.28 vs. 32.3 ± 8.17%, *p* = 0.044) than men (not adjusted for age), although the percentage of patients classified with abnormal values for flow-mediated dilation (FMD) and microvascular ED were not significantly different.

**Table 1:** Clinical characteristics of the HFpEF study sample (*N* = 171) stratified by sex

	Men [*n* = 58]	Women [*n* = 113]	*p*-value
Age [years]	72.2 ± 6.9	68.4 ± 8.8	**0.003**
≤ 60 years	4 (6.9)	16 (14.2)	
60–<70 years	15 (25.9)	40 (35.4)	
70–<80 years	33 (56.9)	47 (41.6)	
≥ 80 years	6 (10.3)	10 (8.9)	
Body weight [kg]	91.9 ± 13.3	78.9 ± 17.0	**< 0.001**
Body fat [%]	26.2 ± 6.13	34.6 ± 6.39	**< 0.001**
Body mass index [kg/m²]	30.0 ± 4.39	30.2 ± 6.16	0.806
Systolic blood pressure [mmHg]	129 ± 13.8	128 ± 13.9	0.569
Diastolic blood pressure [mmHg]	72.9 ± 10.8	75.1 ± 9.88	0.199
Heart rate [bpm]	64.0 ± 10.3	65.4 ± 11.0	0.410
**Medical history**			
NYHA functional class			1.000
II	43 (74.1)	84 (74.3)	
III	15 (25.9)	29 (25.7)	
Time since HF diagnosis [years]	2.00 [0.00;3.50]	1.00 [0.00;3.00]	0.288
Diagnosis > 1 year	38 (65.5)	77 (68.1)	0.862
Atrial fibrillation	17 (29.3)	32 (28.3)	1.000
Chronic kidney disease	18 (31.0)	45 (39.8)	0.337
Coronary heart disease	33 (57.9)	17 (15.9)	**< 0.001**
Previous myocardial infarction	15 (26.3)	11 (9.73)	**0.009**
Diabetes	26 (44.8)	20 (17.9)	**< 0.001**
Hypertension	54 (93.1)	94 (83.2)	0.118
Hyperlipidemia	47 (82.5)	74 (66.1)	**0.040**
Sleep apnea	22 (39.3)	11 (9.91)	**< 0.001**
Smoker			**< 0.001**
Current	4 (6.9)	4 (3.54)	
Ex	36 (62.1)	33 (29.2)	
**Blood parameters**			
Total cholesterol [mg/dL]	170 ± 40.1	206 ± 41.7	**< 0.001**
LDL cholesterol [mg/dL]	103 ± 34.8	127 ± 36.2	**< 0.001**
HDL cholesterol [mg/dL]	49.4 ± 14.1	62.2 ± 15.2	**< 0.001**
Triglycerides [mg/dL]	141 ± 74.7	126 ± 60.2	0.194
NT-proBNP [ng/L]	709 ± 1045	657 ± 1381	0.783
eGFR [mL/min/1.73 m²]	71.2 ± 22.9	74.7 ± 19.9	0.313
**Medication**			
ACE inhibitors	22 (37.9)	31 (27.4)	0.218
Angiotensin receptor blocker	26 (44.8)	48 (42.5)	0.896
Beta blocker	41 (70.7)	72 (63.7)	0.459
Diuretics	33 (56.9)	69 (61.1)	0.718
Aldosterone Antagonists	7 (12.1)	12 (10.6)	0.977
Statins	42 (72.4)	50 (44.2)	**0.001**
Acetylsalicylic acid	34 (58.6)	21 (18.6)	**< 0.001**
**Cardiopulmonary exercise testing**			
VO_2peak_ [ml/min/kg]	19.4 ± 5.54	18.5 ± 5.23	0.300
VO_2peak_ predicted [%]	85.5 ± 21.1	93.5 ± 21.7	**0.022**
Work load [Watt]	119 ± 44.1	93.6 ± 27.8	**< 0.001**
Peak RER	1.09 ± 0.09	1.12 ± 0.11	0.060
Peak heart rate [bpm]	117 ± 26.0	125 ± 25.4	**0.046**
**Vascular assessment**			
FMD [%]	3.52 ± 2.90 [*n* = 19]	5.06 ± 3.06 [*n* = 40]	0.069
Abnormal FMD	7 (36.8)	8 (20.0)	0.207
RHI	1.99 ± 0.60 [*n* = 40]	2.32 ± 0.74 [*n* = 67]	**0.014**
FRHI	0.37 ± 0.41	0.69 ± 0.42	**< 0.001**
Microvascular ED	8 (20.0)	11 (16.4)	0.835
Aix [%]	32.2 ± 8.17 [*n* = 32]	35.9 ± 8.28 [*n* = 61]	**0.044**
PWV [m/s] (corrected*)	11.7 ± 3.32 [*n* = 11]	9.68 ± 1.76 [*n* = 20]	0.080

Data are presented as mean ± SD, median [1st quartile; 3rd quartile] or frequency (%), as appropriate. Variables presented as mean ± SD were compared with independent t-tests, variables presented as median [1st quartile; 3rd quartile] with Mann–Whitney U tests and categorical data with χ2 tests or Fisher’s exact tests

* for carotid femoral distance

ACE, Angiotensin-converting enzyme; Aix, augmentation index; CKD-EPI, Chronic Kidney Disease Epidemiology Collaboration; ED, endothelial dysfunction; eGFR, estimated Glomerular Filtration Rate; FMD, flow-mediated dilation; FRHI, Framingham reactive hyperaemia index; HDL, high-density lipoprotein; HF, heart failure; LDL, low-density lipoprotein; NT-proBNP, N-terminal prohormone of brain natriuretic peptide; NYHA, New York Heart Association; PWV, pulse wave velocity; RER, respiratory exchange ratio; RHI, reactive hyperaemia index; VO2, oxygen consumption

### Metabolites and ratios of the NO pathway – influence of age and sex

**Fig. 1:**
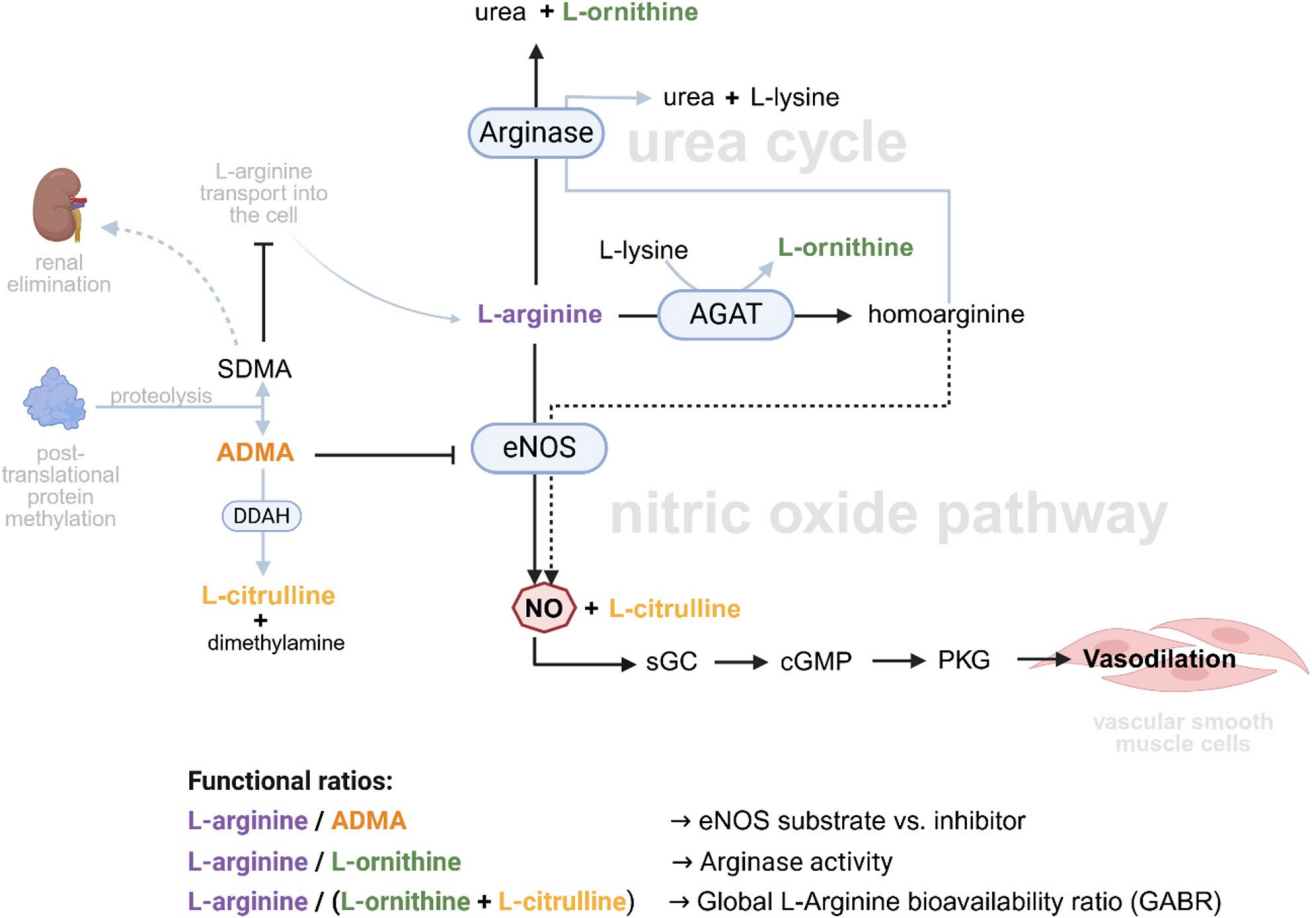
Schematic overview of nitric oxide-related metabolites and ratios. Created in BioRender. Dinges, S. (2026) https://BioRender.com/9it7izq. Abbreviations: ADMA, asymmetric dimethylarginine; AGAT, arginine: glycine amidinotransferase; cGMP, cyclic guanosine monophosphate; DDAH, dimethylarginine dimethylaminohydrolase; eNOS, endothelial nitric oxide synthase; NO, nitric oxide; PKG, protein kinase G; SDMA, symmetric dimethylarginine; sGC, soluble guanylate cyclase

An overview of the investigated metabolites and ratios and their connection within the NO-metabolism is shown in Fig. 1. Median plasma concentrations and ratios are presented for each sex and further stratified by age in Table 2 and visually depicted in Suppl. Figure 1 (A-J). Significant age-related associations were found for SDMA in men (β-coefficient [95% CI]; log-transformed: 0.013 [0.001 to 0.025]; *p* = 0.035) and women (log-transformed: 0.009 [0.003 to 0.015], *p* = 0.002), corresponding to approximately 1.3% and 0.9% higher levels per one-year higher age, respectively, without a significant sex*age interaction (*p* = 0.568). In women, age-related differences were also observed for ADMA (0.003 [0.001 to 0.005] µmol/L, *p* = 0.007) and L-ornithine (0.638 [0.16 to 1.11] µmol/L, *p* = 0.009). No other significant age-related differences or sex*age interactions were observed **(**Table 2**)**.

**Table 2:** Nitric oxide metabolites and ratios stratified by sex and age class (unadjusted)

	Men [*n* = 58]		Women [*n* = 113]		*p*_sex*age_
	Median [1st; 3rd quartile]	β coefficient [95% CI], *p*_age_	Median [1st; 3rd quartile]	β coefficient [95% CI], *p*_age_	
**NO metabolites**					
**L-arginine [µmol/L]**,	**67.9 [58.5;82.0]**	0.017 [−0.69 to 0.72]; *p* = 0.962	**67.7 [59.4;81.9]**	0.182 [−0.20 to 0.57]; *p* = 0.349	0.677
≤60 years	61.3 [52.0;70.8]		66.6 [63.0;74.4]		
60–<70 years	68.4 [63.4;91.3]		71.3 [65.0;82.0]		
70–<80 years	69.5 [58.4;81.7]		66.3 [58.6;79.7]		
≥80 years	63.6 [58.6;74.6]		74.0 [59.0;86.0]		
**L-homoarginine [µmol/L]**	**1.33 [1.00;1.85]**	−0.014 [−0.04 to 0.02]; *p* = 0.340	**1.22 [0.74;1.61]**	−0.012 [−0.03 to 0.00] *p* = 0.104	0.890
≤60 years	1.46 [0.81;2.25]		1.39 [1.02;1.88]		
60–<70 years	1.38 [1.14;1.80]		1.29 [0.72;1.57]		
70–<80 years	1.40 [1.02;1.91]		1.06 [0.78;1.64]		
≥80 years	1.14 [0.87;1.55]		1.03 [0.68;1.32]		
**ADMA [µmol/L]**	**0.62 [0.58;0.68]**	0.003 [−0.001 to 0.006]; *p* = 0.160	**0.61 [0.55;0.66]**	**0.003** **[0.001 to 0.005]** *p* = **0.007**	0.936
≤60 years	0.62 [0.61;0.65]		0.61 [0.54;0.64]		
60–<70 years	0.59 [0.53;0.62]		0.57 [0.52;0.63]		
70–<80 years	0.66 [0.62;0.72]		0.61 [0.57;0.68]		
≥80 years	0.61 [0.58;0.64]		0.67 [0.64;0.71]		
**SDMA [µmol/L]**	**0.72 [0.58;0.82]**	* 0.013 **[0.001 to 0.025]; p = 0.035**	**0.60 [0.52;0.72]**	* 0.009 **[0.003 to 0.015]** *p* = **0.002**	0.568
≤60 years	0.66 [0.57;0.95]		0.52 [0.48;0.60]		
60–<70 years	0.59 [0.56;0.72]		0.59 [0.53;0.70]		
70–<80 years	0.75 [0.63;0.92]		0.62 [0.55;0.75]		
≥80 years	0.79 [0.65;0.82]		0.77 [0.68;1.03]		
**L-citrulline [µmol/L]**	**42.9 [32.3;51.1]**	0.662 [−0.00 to 1.32]; *p* = 0.05	**40.3 [33.0;47.5]**	0.257 [−0.00 to 0.52] *p* = 0.053	0.193
≤60 years	34.7 [23.0;45.3]		37.2 [31.1;45.7]		
60–<70 years	34.6 [27.6;50.7]		39.5 [34.1;45.9]		
70–<80 years	42.7 [32.4;52.2]		40.3 [33.9;46.2]		
≥80 years	44.6 [37.9;49.3]		53.1 [42.4;59.6]		
**L-lysine [µmol/L]**	**191 [172;213]**	−0.495 [−1.56 to 0.57]; *p* = 0.357	**183 [164;205]**	0.413 [−0.26 to 1.08] *p* = 0.224	0.172
≤60 years	184 [175;195]		177 [151;211]		
60–<70 years	199 [171;221]		182 [165;204]		
70–<80 years	195 [174;213]		182 [164;205]		
≥80 years	176 [164;182]		195 [185;199]		
**L-ornithine [µmol/L]**	**71.9 [59.0;87.6]**	0.245 [−0.62 to 1.11]; *p* = 0.573	**71.3 [59.6;82.4]**	**0.638** **[0.16 to 1.11]** *p* = **0.009**	0.422
≤60 years	61.4 [55.6;67.9]		63.9 [59.5;73.7]		
60–<70 years	68.9 [61.6;85.1]		66.5 [56.0;81.1]		
70–<80 years	81.6 [66.1;89.4]		72.2 [62.6;82.0]		
≥80 years	58.0 [54.4;74.4]		87.9 [76.8;108]		
**Ratios**					
**L-arginine/ADMA ratio**	**104 [85.3;134]**	−0.459 [−1.66 to 0.75]; *p* = 0.449	**117 [97.6;131]**	−0.252 [−0.92 to 0.41] *p* = 0.454	0.762
≤60 years	99.1 [80.7;117]		111 [97.0;132]		
60–<70 years	124 [92.6;153]		123 [109;135]		
70–<80 years	102 [85.1;126]		109 [94.8;124]		
≥80 years	104 [95.1;118]		98.2 [89.9;132]		
**L-arginine/L-ornithine ratio**	**0.96 [0.76;1.10]**	−0.002 [−0.01 to 0.01], *p* = 0.673	**1.00 [0.84;1.21]**	−0.005 [−0.01 to 0.00], *p* = 0.141	0.674
≤60 years	1.00 [0.93;1.04]		1.03 [0.95;1.23]		
60–<70 years	1.03 [0.90;1.08]		1.15 [0.85;1.25]		
70–<80 years	0.86 [0.75;1.07]		0.98 [0.78;1.12]		
≥80 years	1.03 [0.81;1.33]		0.83 [0.75;0.99]		
**GABR**	**0.60 [0.52;0.71]**	−0.005 [−0.01 to 0.00]; *p* = 0.078	**0.65 [0.55;0.75]**	−0.003 [−0.01 to 0.00] *p* = 0.153	0.552
≤60 years	0.71 [0.63;0.74]		0.66 [0.58;0.80]		
60–<70 years	0.65 [0.59;0.75]		0.70 [0.55;0.80]		
70–<80 years	0.57 [0.50;0.64]		0.63 [0.53;0.69]		
≥80 years	0.59 [0.53;0.69]		0.52 [0.44;0.65]		

Data is presented as median [1st; 3rd quartile]

Sample size of age groups (men/women): ≤60 years (4/16); 60–<70 years (15/40); 70–<80 years (33/47), ≥ 80 years (6/10)

Results show the regression coefficient β for age and the corresponding 95% confidence interval, or the p-value for the interaction of sex*age

* Model did not meet model assumptions and therefore, data was log-transformed

Unadjusted regression analysis (*Model 1*) showed significant sex-related differences for hArg (−0.24 [−0.46 to −0.01] µmol/L, *p* = 0.043) and SDMA (−0.11 [−0.20 to −0.03] µmol/L, *p* = 0.010), both with lower plasma concentrations in women **(**Table 3**)**. The sex differences remained significant for both metabolites after adjusting for age (*Model 2*). For hArg, a significant sex difference was no longer present after the adjustment for BMI and HDL cholesterol (*Model 4*) until full adjustment. For SDMA, the direction of the association with sex was consistent across all models. Although statistical significance was not reached after adjustment for %-predicted VO2peak (*Model 3*), effect estimates were of similar magnitude across models and reached statistical significance in the more comprehensively adjusted models (*Models 4–6*). No significant sex differences were observed for the remaining metabolites (L-arginine, ADMA, L-citrulline, L-lysine and L-ornithine) or ratios (L-arginine/ADMA, L-arginine/L-ornithine ratio, and GABR) across all models.

**Table 3:** Results of the multivariable regression analysis

Marker	Model 1 (unadjusted)	Model 2	Model 3	Model 4	Model 5	Model 6
**L-arginine [µmol/L]**	1.31 (−4.44 to 7.06), *p* = 0.654	1.85 (−4.05 to 7.74), *p* = 0.537	1.62 (−4.35 to 7.60), *p* = 0.593	1.85 (−4.66 to 8.36), *p* = 0.576	1.36 (−5.60 to 8.32), *p* = 0.700	0.8 (−6.25 to 7.85), *p* = 0.823
**L-homoarginine** **[µmol/L]**	−0.24 (−0.46 to −0.01), *p* **= 0.043**	−0.28 (−0.51 to −0.05), *p* **= 0.017**	−0.30 (−0.53 to −0.07), *p* **= 0.012**	−0.23 (−0.48 to 0.02), *p* = 0.077	−0.18 (−0.45 to 0.09), *p* = 0.192	−0.20 (−0.47 to 0.07), *p* = 0.150
**ADMA [µmol/L]**	−0.03 (−0.06 to 0.003), *p* = 0.076	−0.02 (−0.05 to 0.01), *p* = 0.261	−0.01 (−0.04 to 0.02), *p* = 0.528	−0.01 (−0.04 to 0.02), *p* = 0.494	−0.01 (−0.05 to 0.02), *p* = 0.470	−0.02 (−0.05 to 0.02), *p* = 0.350
**SDMA [µmol/L]**	−0.11 (−0.20 to −0.03), p = **0.010**	−0.09 (−0.18 to 0), p = **0.043**	−0.06 (−0.14 to 0.02), *p* = 0.152	−0.09 (−0.18 to 0), *p* **= 0.045**	−0.10 (−0.17 to −0.04), *p* **= 0.002**	−0.11 (−0.17 to −0.04), *p* **= 0.002**
**L-citrulline [µmol/L]**	−0.82 (−5.42 to 3.77), *p* = 0.724	0.52 (−4.10 to 5.14), *p* = 0.824	1.45 (−3.13 to 6.04), *p* = 0.533	−0.51 (−5.43 to 4.41), *p* = 0.838	−1.27 (−6.04 to 3.49), *p* = 0.598	−1.25 (−5.99 to 3.50), *p* = 0.605
**L-lysine [µmol/L]**	−7.26 (−16.90 to 2.38), *p* = 0.139	−6.53 (−16.41 to 3.36), *p* = 0.194	−7.35 (−17.34 to 2.64), *p* = 0.148	−4.52 (−15.26 to 6.22), *p* = 0.407	−1.23 (−12.59 to 10.12), *p* = 0.831	−1.45 (−12.76 to 9.86), *p* = 0.800
**L-ornithine [µmol/L]**	−1.97 (−9.20 to 5.24), *p* = 0.589	0.07 (−7.20 to 7.34), *p* = 0.984	0.17 (−7.2 to 7.54), *p* = 0.964	−0.56 (−8.63 to 7.51), *p* = 0.892	−0.03 (−8.65 to 8.59), *p* = 0.994	0.77 (−7.92 to 9.47), *p* = 0.861
**L-arginine/ADMA ratio**	7.33 (−2.61 to 17.26), *p* = 0.147	6.18 (−3.99 to 16.36), *p* = 0.232	4.46 (−5.70 to 14.61), *p* = 0.388	4.83 (−6.24 to 15.90), *p* = 0.390	4.15 (−7.42 to 15.72), *p* = 0.479	3.95 (−7.81 to 15.70), *p* = 0.508
**L-arginine/L-ornithine ratio**	0.06 (−0.04 to 0.16), *p* = 0.225	0.05 (−0.06 to 0.15), *p* = 0.389	0.04 (−0.06 to 0.15), *p* = 0.447	0.05 (−0.06 to 0.17), *p* = 0.356	0.04 (−0.08 to 0.16), *p* = 0.478	0.03 (−0.09 to 0.15), *p* = 0.645
**GABR**	0.04 (−0.02 to 0.09), *p* = 0.235	0.02 (−0.04 to 0.08), *p* = 0.469	0.02 (−0.04 to 0.07), *p* = 0.608	0.03 (−0.04 to 0.09), *p* = 0.398	0.03 (−0.04 to 0.09), *p* = 0.405	0.02 (−0.05 to 0.09), *p* = 0.523

Results show the regression coefficient β for sex and the corresponding 95% confidence interval

-ß coefficient: lower values in women

+ß coefficient: higher values in women

Model 1: unadjusted

Model 2: Model 1 + age

Model 3: Model 2 + %-predicted VO2peak

Model 4: Model 3 + BMI, HDL cholesterol

Model 5: Model 4 + comorbidities (diabetes, hypertension, coronary artery disease, atrial fibrillation, eGFR)

Model 6: Model 5 + ACE inhibitors, angiotensin receptor blocker, diuretics, and statins

### Sex-stratified NO metabolite patterns

**Fig. 2:**
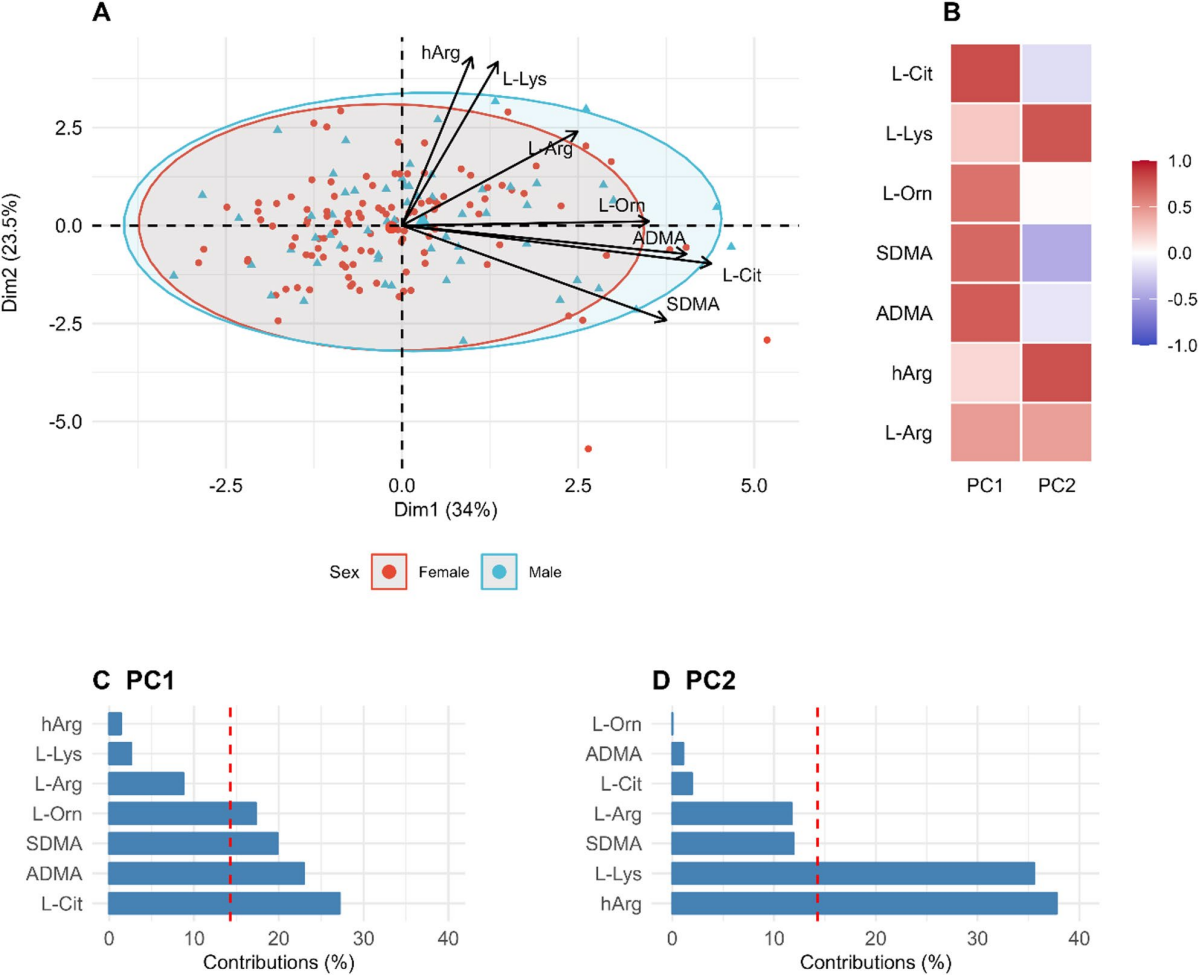
Principal component analysis of NO-related metabolites in HFpEF. (**A**) Biplot of the first two principal components (PC1, PC2) showing the distribution of female (red) and male (blue) patients with HFpEF with 95% confidence ellipses. Arrows indicate loading vectors of individual metabolites. (**B**) Heatmap of metabolite correlations for the first two PCs. (**C**–**D**) Variable contributions to PC1 and PC2. The dashed line indicates the expected average contribution

To explore sex-stratified multivariate patterns across NO-related metabolites, a PCA (Fig. 2) was conducted. The first two principal components explained 57.5% of the total variance, with PC1 accounting for 34% and PC2 for 23.5%. PC1 was mainly driven by L-citrulline, ADMA, SDMA and L-ornithine (Fig. 2B **+ C**), whereas PC2 was predominantly influenced by hArg and L-lysine (Fig. 2B **+ D**). The visual inspection of the PCA biplot, including sex-stratified confidence ellipses, revealed a substantial overlap between men and women with no distinct clustering of the circulating NO-related metabolic profile.

## Discussion

In this work, plasma concentrations of a broad panel of NO-related amino acids, methylated arginines, and functional ratios were analysed in patients with HFpEF, stratified by sex and age. The main findings of this study were: (i) age-related associations with NO metabolites were modest and metabolite specific, without evidence of sex-age interactions; (ii) significant sex differences were observed for SDMA and hArg, although the association with hArg was largely attenuated after adjustment for clinical covariates; and (iii) multivariate analysis did not reveal distinct sex-related NO-related metabolic patterns.

Overall, our findings do not support the hypothesis that sex differences in HFpEF pathophysiology are largely reflected in circulating NO metabolites. Most metabolites and ratios did not differ between men and women, a finding further supported by the substantial overlap of global metabolic profiles in multivariate analyses. While sex differences in hArg concentrations vanished after adjustment, SDMA showed the most consistent differences between sexes.

SDMA limits intracellular NO production indirectly by competing with L-arginine for cellular transport and is predominantly eliminated by renal excretion [21, 22]. Beyond its role as a marker of kidney function, elevated SDMA concentrations have been linked to CV disease, all-cause mortality [9], and long-term decline in renal function [23], highlighting its relevance as a biomarker of cardiometabolic risk. The sex-related difference in SDMA persisted despite comparable renal function and adjustment for clinical covariates, suggesting that this difference is not solely explained by kidney function or comorbidity burden. Higher SDMA concentrations in men are consistent with results from previously reported reference ranges derived from a healthy cohort [11] and findings from the population-based Dallas Heart Study [24]. However, absolute SDMA concentrations were substantially higher in our HFpEF cohort, likely reflecting the advanced disease and comorbidity burden characteristic of HFpEF. Recent findings show that SDMA is strongly linked to the activity of alanine-glyoxylate-aminotransferase 2 (AGXT2), an enzyme primarily expressed in the kidneys [25]. Renal dysfunction may thus be one primary comorbidity in HFpEF that is mechanistically linked to high SDMA concentrations.

We also observed lower circulating hArg concentrations in women compared to men with HFpEF, again consistent with sex-specific reference values derived from healthy populations [10, 11] and studies in healthy older adults [26]. However, this association was attenuated after adjustment for clinical covariates BMI and HDL cholesterol (*Model 4*). A comparable pattern has been reported in the Dallas Heart Study, where adjustment for several measures of body composition even reversed the initial sex difference [24]. In the same cohort, strong correlations of hArg with anthropometric measures such as BMI and lean mass were described, and hArg was positively and independently associated with BMI [27]; however, we did not find such correlations in our HFpEF sample [15]. The enzyme arginine:glycine amidinotransferase (AGAT) synthesises hArg from L-lysine and L-arginine, but also catalyses the first step of creatine biosynthesis [7], thereby linking hArg metabolism and muscle energy homeostasis. In heart failure, both creatine and hArg levels are reduced [28, 29]. In our cohort, hArg concentrations were lower than in comparable populations [10, 11, 26], and low concentrations of hArg were previously shown to be linked to adverse CV outcomes [30].

In all other amino acids, ADMA and composite ratios, we observed no significant differences. In general, evidence for differences in amino acids between men and women is limited and inconsistent, and comparisons across studies are hampered by the lack of sex-stratified reporting, different methods, age distributions and small sample sizes [31]. A study in healthy older adults of similar age to our cohort also found no sex differences in L-arginine, L-ornithine, L-citrulline, ADMA and SDMA, but higher hArg levels in men [26]. Circulating amino acid concentrations reflect a complex interplay of dietary intake, endogenous synthesis, metabolic turnover (e.g. urea cycle or NO pathway) and renal regulation [7]. To account for this complexity, functional ratios have been proposed to capture pathway-related alterations and have been associated with CV outcomes in several clinical settings [32, 33]. However, sex-stratified analyses remain scarce. A statistically significant lower L-arginine/ADMA ratio in women has been reported in a large population-based cohort, also for L-arginine, ADMA, and SDMA; however, absolute differences were small [34]. 

For ADMA, previous studies reported heterogeneous sex associations with higher levels in healthy men compared to healthy women [11] or modulation by menopausal status in women [35]. Although ADMA concentrations commonly display a narrow range [35], even small increases have been linked to adverse CV outcome [9, 36, 37]. In line with our findings, the analysis of the Dallas Heart Study showed no sex difference in unadjusted analysis and in the first adjusted models including demographics and cardiometabolic risk factors. However, after adjusting for body composition and left ventricular mass, men showed higher ADMA levels than women [24]. In another study in patients with coronary artery disease, ADMA levels were neither associated with sex nor age but were influenced by smoking, diabetes and disease severity [38]. 

Hormonal influences have been proposed as contributors to sex differences, as oestrogen has been shown to enhance ADMA degradation via dimethylarginine dimethylaminohydrolase (DDAH) [39] and to modulate AGAT expression and hArg synthesis under conditions of high hormonal exposure [40]. However, our female HFpEF patients were predominantely post-menopausal. Notably, the effect of age itself may also play a role independent of sex. Previously reported age-related trajectories differ between metabolites and sexes, including declining hArg in both men and women [10] or only in women [11], age-related increases of SDMA in men only [11] or in both sexes [41], and increases in ADMA for men and women [11, 35]. In contrast to healthier populations with broader age ranges, associations in our HFpEF cohort were modest and metabolite-specific without significant sex–age interactions.

Experimental and pre-clinical studies provide increasing evidence on sex-related differences in endothelial cells and vascular biology independently of hormonal control [42]. While experimental work allows the assessment of tissue-specific NO signalling, endothelial enzyme activity, and myocardial or vascular alterations, the present study measured systemic circulating metabolites in the blood of patients with HFpEF. However, local impairment of NO bioavailability or differences in male and female endothelial cells may not necessarily translate into differences in circulating plasma concentrations. This translational complexity is further amplified by the marked phenotypic heterogeneity of clinical presentation in patients, reflecting different combinations of risk factors, comorbidities and pathophysiologic contributors. This is supported by previously observed differences in NO-related metabolites between HFrEF and HFpEF, suggesting that circulating NO-related biomarker profiles may vary across HF phenotypes, but are difficult to disentangle from disease aetiology, ischemic burden, and comorbidities [15]. In addition, pharmacological treatment itself needs to be considered, as several CV medications may influence the NO pathway and the measured metabolites. In particular, statins [43], and other drug classes as well as CV polytherapy may also affect circulating ADMA levels [44]. Although our multivariable models adjusted for several clinically relevant variables related to NO metabolism, women and men still differed in several baseline characteristics that may have influenced the observed associations. Consequently, observed differences between women and men should not be interpreted as reflecting biological sex alone, but may be influenced by underlying HFpEF phenotypes and related treatment.

Taken together, the observed sex-related differences in SDMA and hArg partly mirror patterns reported in healthy or population-based cohorts. However, the overall metabolite profile in HFpEF appears shifted towards a less favourable NO-related biomarker pattern. Thus, previously described differences observed in healthy populations may persist in HFpEF, but were not clearly enhanced despite known sex-related differences in HFpEF pathophysiology.

### Perspective and significance

From a broader perspective, our findings support the continued importance of sex-stratified analyses in HFpEF biomarker research. However, biological sex should not be interpreted in isolation, as sex-related differences in circulating NO-related metabolites may partly be explained by HFpEF phenotype, including differences in renal function, body composition, vascular dysfunction, comorbidities, and medication use. Future studies should therefore combine sex-stratified approaches with detailed phenotypic characterization to better determine whether NO-related metabolites reflect biological sex, specific HFpEF phenotypes, disease progression, or risk beyond established clinical biomarkers.

### Limitations

Several limitations need to be considered. This study is an exploratory secondary analysis from a previously performed clinical trial. Therefore, the analysis was not based on an a priori sample size calculation and may have been underpowered to detect modest sex-related differences in circulating NO-related metabolites, particularly given the unequal distribution of men and women. Furthermore, the cross-sectional design and the statistical approach do not allow causal conclusions and the observed findings should therefore be interpreted as associations. Although multivariable models were adjusted for major clinical characteristics, residual confounding by differences in HFpEF phenotypes and medication cannot be excluded.

The analysed patients are from an exercise trial and therefore had to be clinically stable, which limits generalisability. Patients were instructed to be in a fasting state for blood sampling; however, information on dietary intake was not assessed. Although several measures were taken to ensure high sample quality, potential effects of long-term storage (up to 5 years between blood draw and analysis) cannot be fully excluded. However, all samples were handled and stored under comparable conditions and analysed centrally in the same laboratory, making substantial differential bias between women and men unlikely. BMI was used to adjust for obesity, as more robust measures of body composition and fat distribution were not consistently available across all patients and study sites. Moreover, vascular function measurements were available only in a subset of participants, precluding adequately adjusted sex-stratified analyses.

Finally, our findings should be interpreted as exploratory and hypothesis-generating for future research and require external validation in larger, well-phenotyped HFpEF cohorts. Nevertheless, to our knowledge, this study provides one of the most comprehensive sex-stratified assessments of NO-related amino acids, methylated arginines, and functional ratios in a well-characterized HFpEF cohort.

## Conclusion

In patients with HFpEF, sex-related differences in circulating NO metabolites were limited to individual metabolites, particularly higher SDMA in men, whereas the overall metabolic profile showed substantial overlap between women and men. These findings suggest that circulating NO-related metabolites in HFpEF should be interpreted in the context of overall disease and phenotype burden rather than biological sex alone.

## Supplementary Information

Supplementary Material 1.

## Data Availability

The clinical data and metabolite data are not publicly available due to privacy restrictions. However, upon request to the corresponding author, aggregated data may be shared. All analyses were performed via standard statistical software and packages as described in the methods section.

## Abbreviations

ACE: Angiotensin-converting enzyme
ADMA: asymmetric dimethylarginine
AGAT: arginine: glycine amidinotransferase
Aix: Augmentation index
BMI: Body mass index
CI: Confidence interval
CV: Cardiovascular
DDAH: Dimethylarginine dimethylaminohydrolase
ED: Endothelial dysfunction
eGFR: Estimated glomerular filtration rate
eNOS: Endothelial nitric oxide synthase
FMD: Flow-mediated dilation
FRHI: Framingham reactive hyperaemia index
GABR: Global arginine bioavailability ratio
hArg: Homoarginine
HDL: High density lipoprotein
HFpEF: Heart failure with preserved ejection fraction
L-Arg: L-arginine
LDL: Low density lipoprotein
NO: Nitric oxide
NYHA: New York Heart Association
PCA: Principal component analysis
PWV: Pulse wave velocity
RHI: Reactive hyperaemia index
SDMA: Symmetric dimethylarginine
VO_2peak_: Peak oxygen consumption
