# Correction: Circulating nitric oxide pathway metabolites in heart failure with preserved ejection fraction: a sex-stratified cross-sectional analysis

**DOI:** 10.1186/s13293-026-00944-3

**Published:** 2026-07-06

**Authors:** Sophia Marie-Theres Dinges, Edzard Schwedhelm, Flavia Baldassarri, Bernhard Haller, Andreas B. Gevaert, Rainer Böger, Ephraim B. Winzer, Frank Edelmann, Ulrik Wisløff, Volker Adams, Burkert Pieske, Emeline M. Van Craenenbroeck, Martin Halle, Susanna M. Hofmann, Stephan Mueller-Stegner

**Affiliations:** 1 TUM School of Medicine and Health, Department for Preventive Sports Medicine and Sports Cardiology, Technical University of Munich, TUM University Hospital, Munich, Germany; 2 DZHK (German Centre for Cardiovascular Research), Partner Site Munich Heart Alliance, Munich, Germany; 3 Research Group “Women and Diabetes”, Institute for Diabetes and Regeneration, Helmholtz Center Munich - German Research Center for Environmental Health, Neuherberg, Germany; 4 Institute of Clinical Pharmacology and Toxicology, University Medical Center Hamburg- Eppendorf, Hamburg, Germany; 5 DZHK (German Centre for Cardiovascular Research), Partner Site North, Hamburg, Germany; 6 TUM School of Medicine and Health, Institute of AI and Informatics in Medicine,, Technical University of Munich, Munich, Germany; 7 Department of Cardiology, Antwerp University Hospital, Edegem, Belgium; 8 Research Group Cardiovascular Diseases, GENCOR Department, University of Antwerp, Antwerp, Belgium; 9 Department of Internal Medicine/Cardiology, Heart Center, University Clinic, Technische Universität Dresden, Dresden, Germany; 10 Department of Cardiology, Angiology and Intensive Care Medicine, Deutsches Herzzentrum der Charité (DHZC) - Medical Heart Centre of Charité and German Heart Institute Berlin, Berlin, Germany; 11 Charité - Universitätsmedizin Berlin, Corporate Member of Freie Universität Berlin, Humboldt Universität zu Berlin, Berlin, Germany; 12 DZHK (German Centre for Cardiovascular Research), Partner Site Berlin, Berlin, Germany; 13 Department of Circulation and Medical Imaging, Faculty of Medicine and Health SciencesDivision of Cardiology, Department of Internal Medicine, Norwegian University of Science and TechnologyUniversity Medicine Rostock, Trondheim, Norway; 14 Division of Cardiology, Department of Internal Medicine, UniversityMedicine Rostock, Rostock,, Germany; 15 Department of Medicine IV, University Hospital, LMU Munich, Munich, Germany; 16 German Center for Diabetes Research (DZD), Neuherberg, Germany

**Correction: Biology of Sex Differences (2026) 17:120**

10.1186/s13293-026-00940-7

Following publication of the original article [1], the authors reported a typesetting error, whereby the footnote for Table 3 was erroneously omitted.

The footnote for Table 3 is given below and the original article [1] has been corrected. The publishers apologise for this error.

Results show the regression coefficient β for sex and the corresponding 95% confidence interval.

-ß coefficient: lower values in women.

+ß coefficient: higher values in women.

Model 1: unadjusted.

Model 2: Model 1 + age.

Model 3: Model 2 + %-predicted VO2peak.

Model 4: Model 3 + BMI, HDL cholesterol.

Model 5: Model 4 + comorbidities (diabetes, hypertension, coronary artery disease, atrial fibrillation, eGFR).

Model 6: Model 5 + ACE inhibitors, angiotensin receptor blocker, diuretics, and statins.
