# Supplementary material for: Circulating nitric oxide pathway metabolites in heart failure with preserved ejection fraction: a sex-stratified cross-sectional analysis
Source: Biol Sex Differ. 2026 Jun 15;17:120. doi: 10.1186/s13293-026-00940-7 (PMC13270708; doi:10.1186/s13293-026-00940-7)
Supplement: Supplementary file 1 — Supplementary Material 1. [file 13293_2026_940_MOESM1_ESM.docx]

**Supplementary Material**

**Suppl. Table 1** Imputed values for covariates used in regression models

| **Parameter** | **Missing values (%)** |
| --- | --- |
| Sex | 0 (0) |
| Age | 0 (0) |
| %pred. VO2peak | 0 (0) |
| BMI [kg/m²] | 0 (0) |
| HDL-cholesterol [mg/dL] | 1 (0.6) |
| Diabetes | 1 (0.6) |
| Hypertension | 0 (0) |
| Coronary artery disease | 7 (4.1) |
| Atrial fibrillation | 0 (0) |
| eGFR [mL/min/1.73 m²] | 1 (0.6) |
| ACE inhibitors | 0 (0) |
| Angiotensin receptor blockers | 0 (0) |
| Diuretics | 0 (0) |
| Statins | 0 (0) |
| Data is shown as number (%). | |

**Suppl. Figure 1** Metabolites (A-G) and ratios (H-J) stratified by sex and in relation to age

Plots A-J include all 171 analysed patients (n= 58 men, n= 113 women)


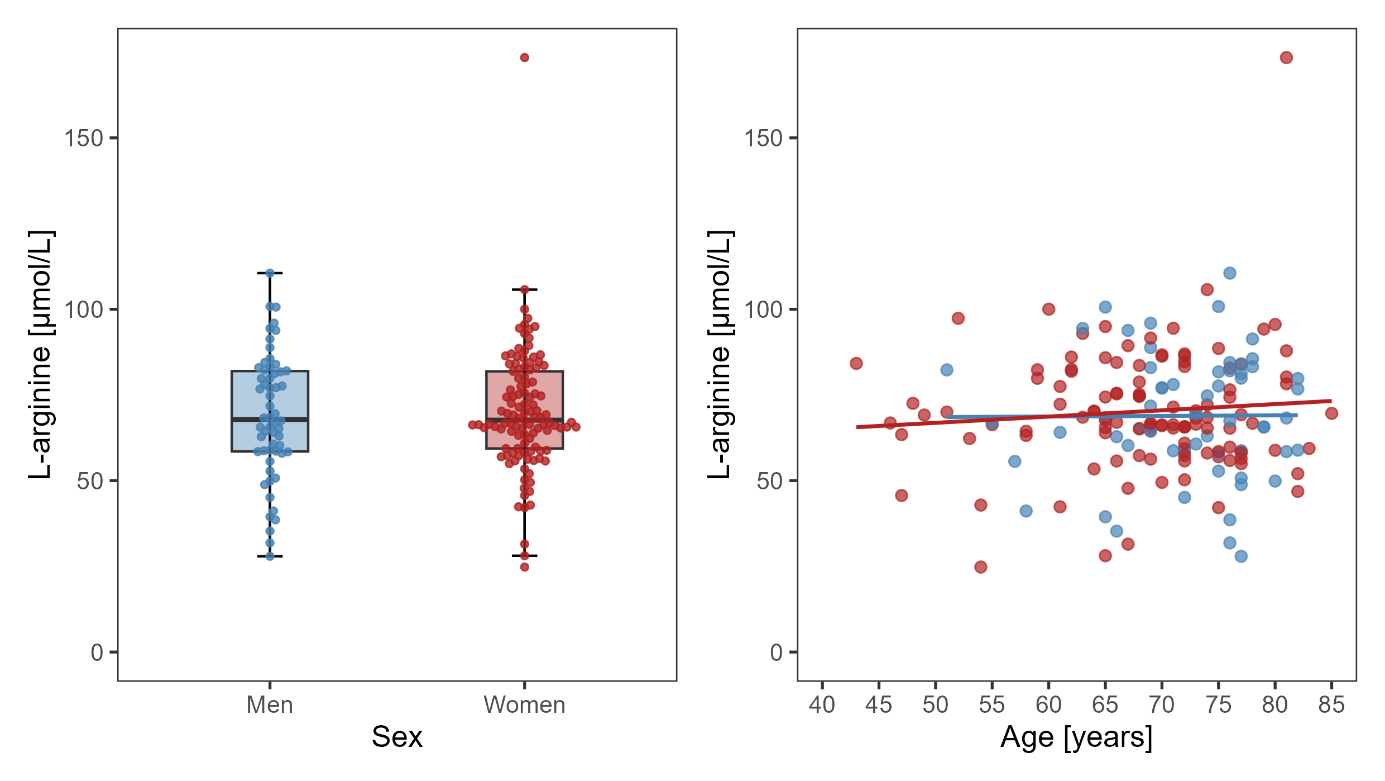


**B)**

**
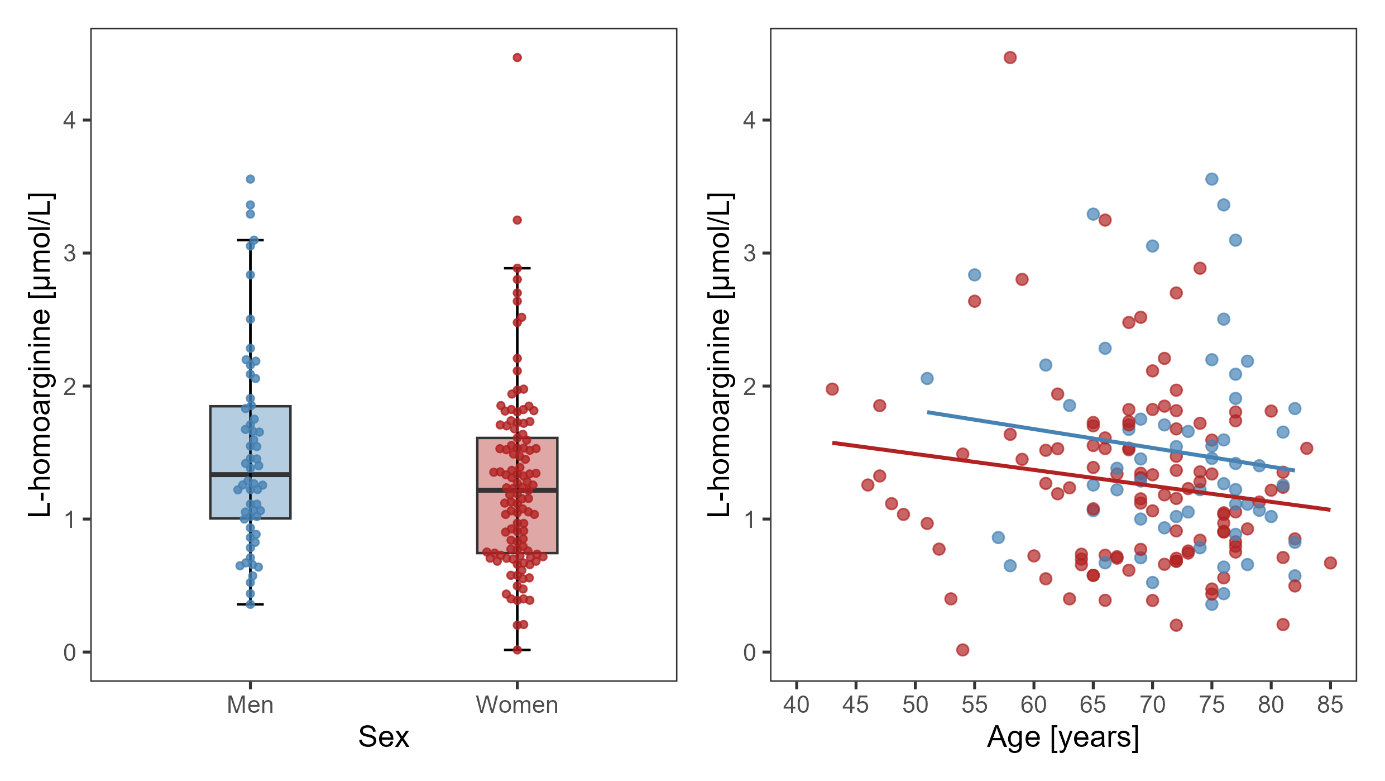
**

**C)**

**
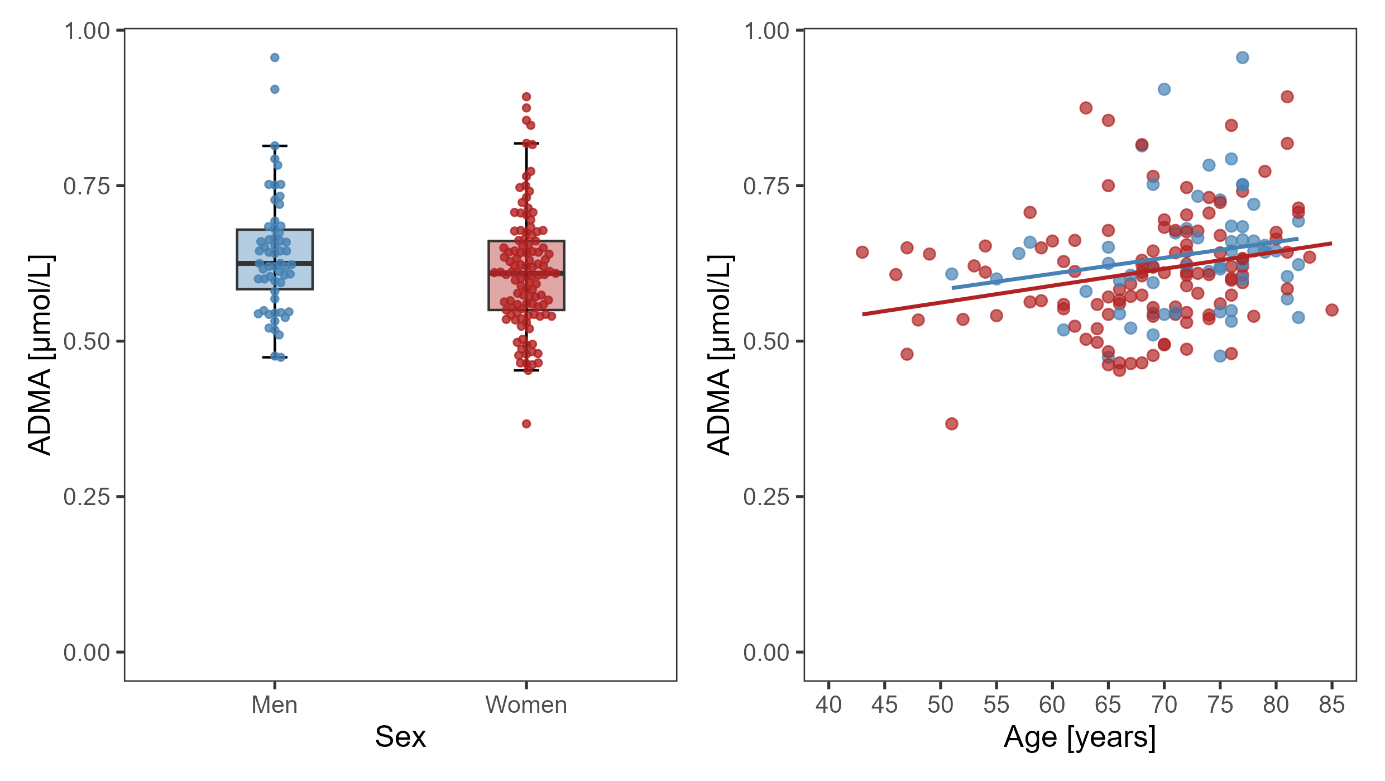
**

**D)
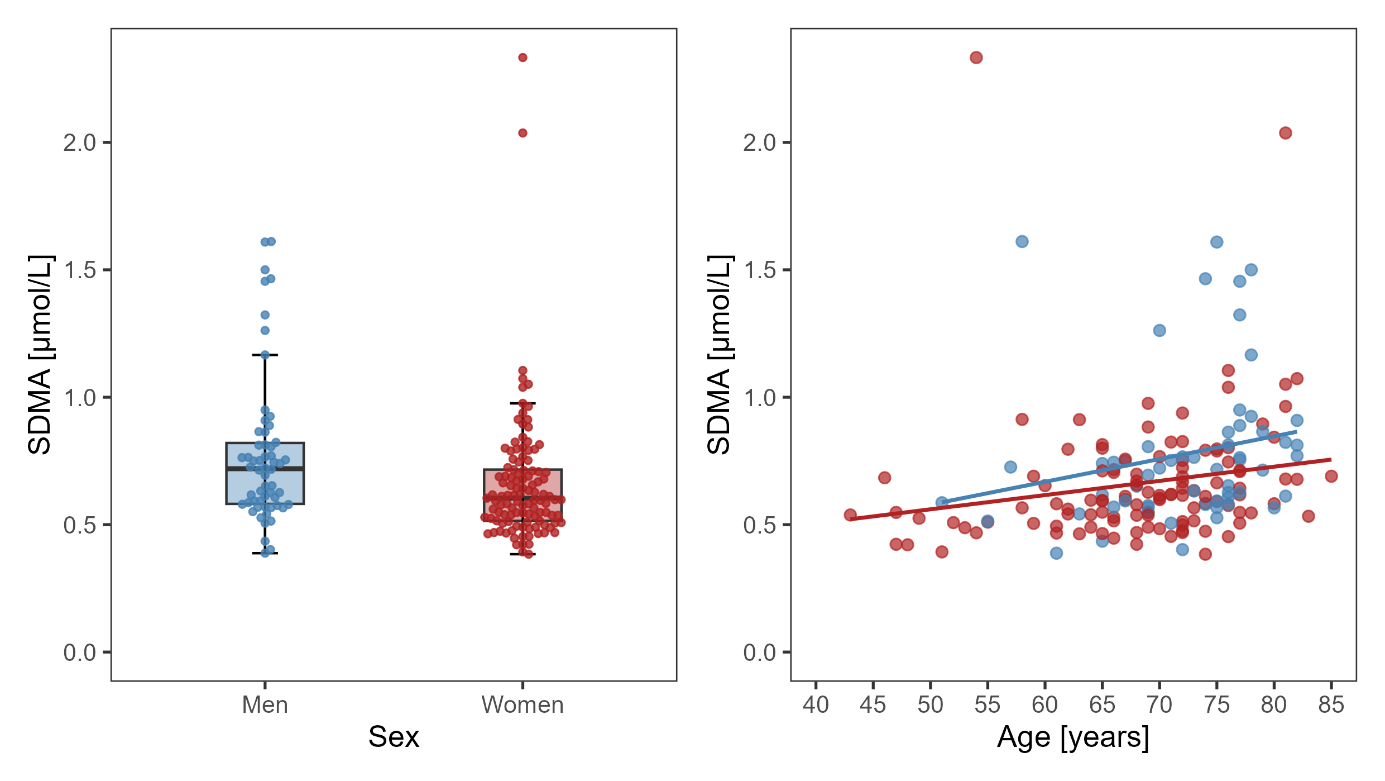
**

**E)
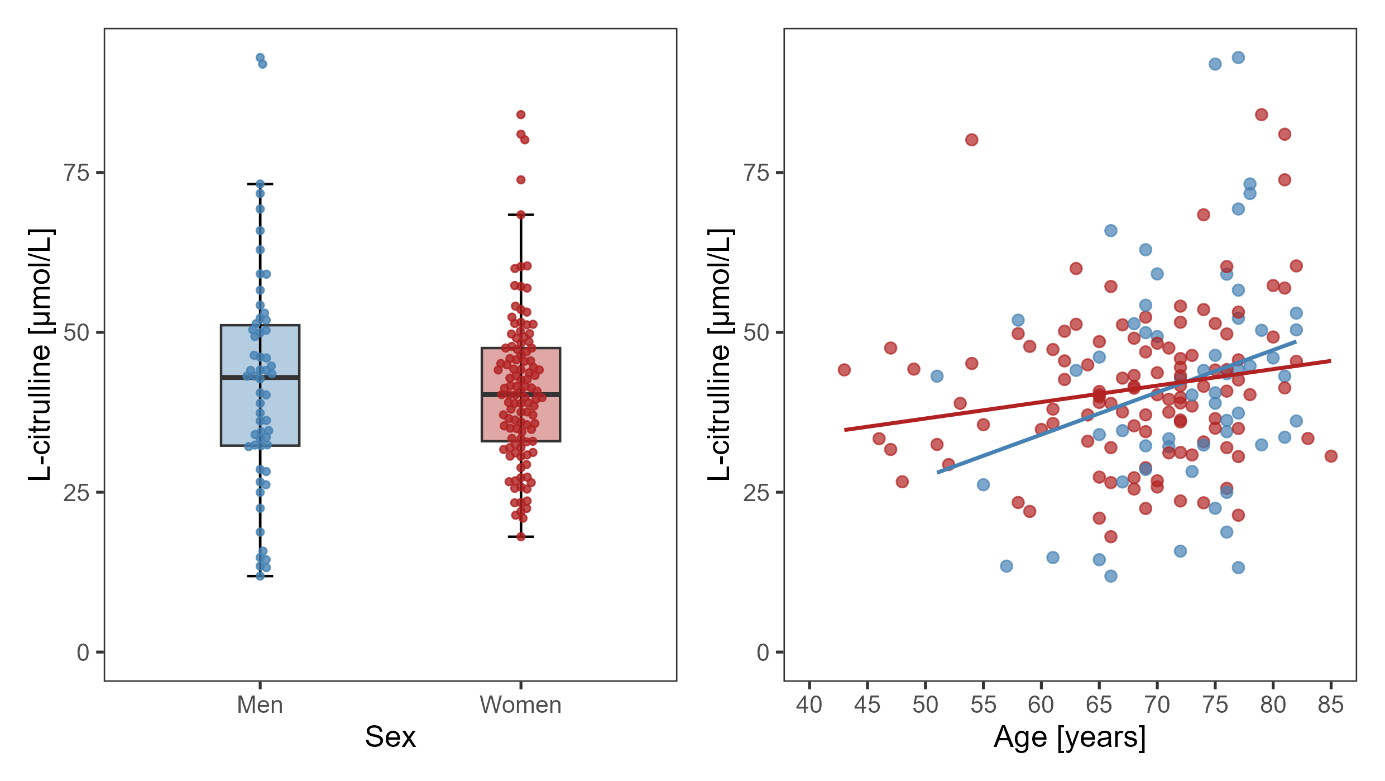
**

**F)
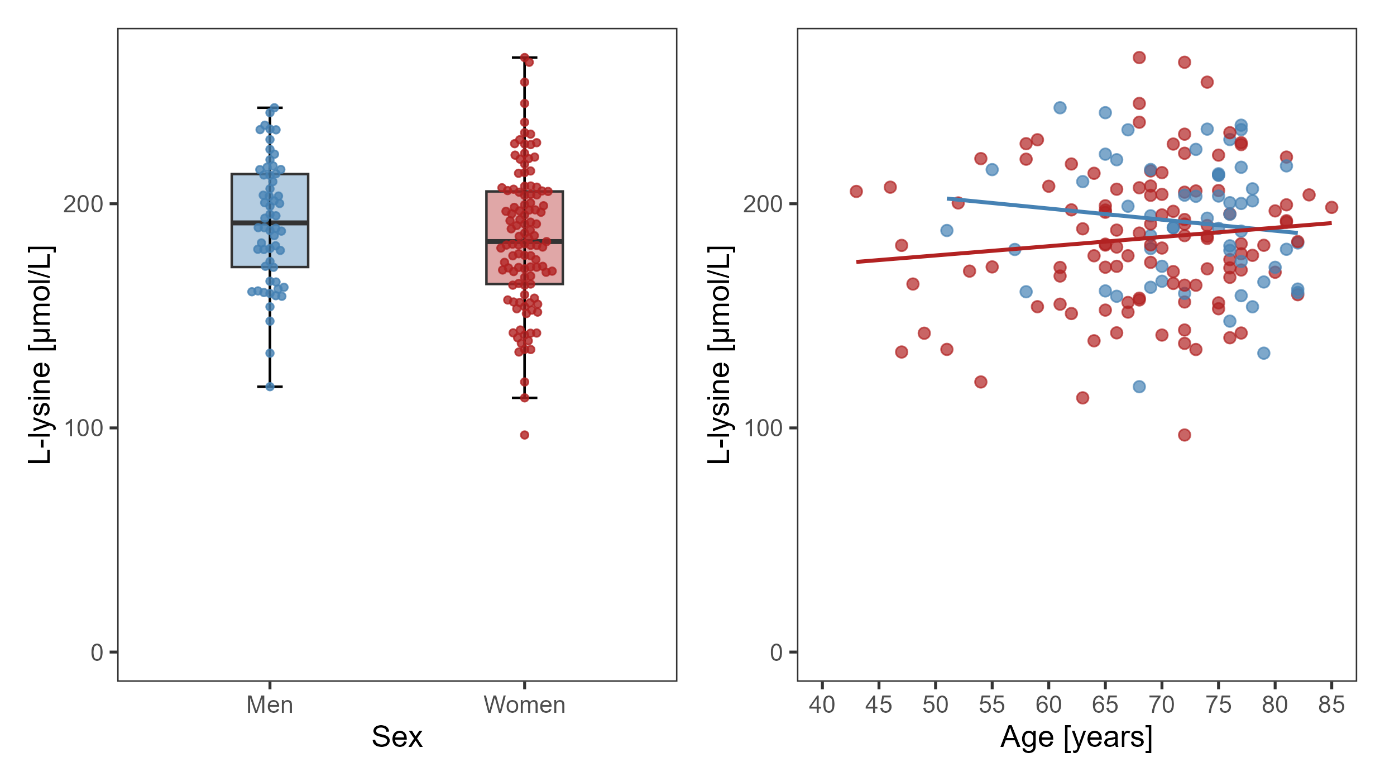
**

**G)**

**
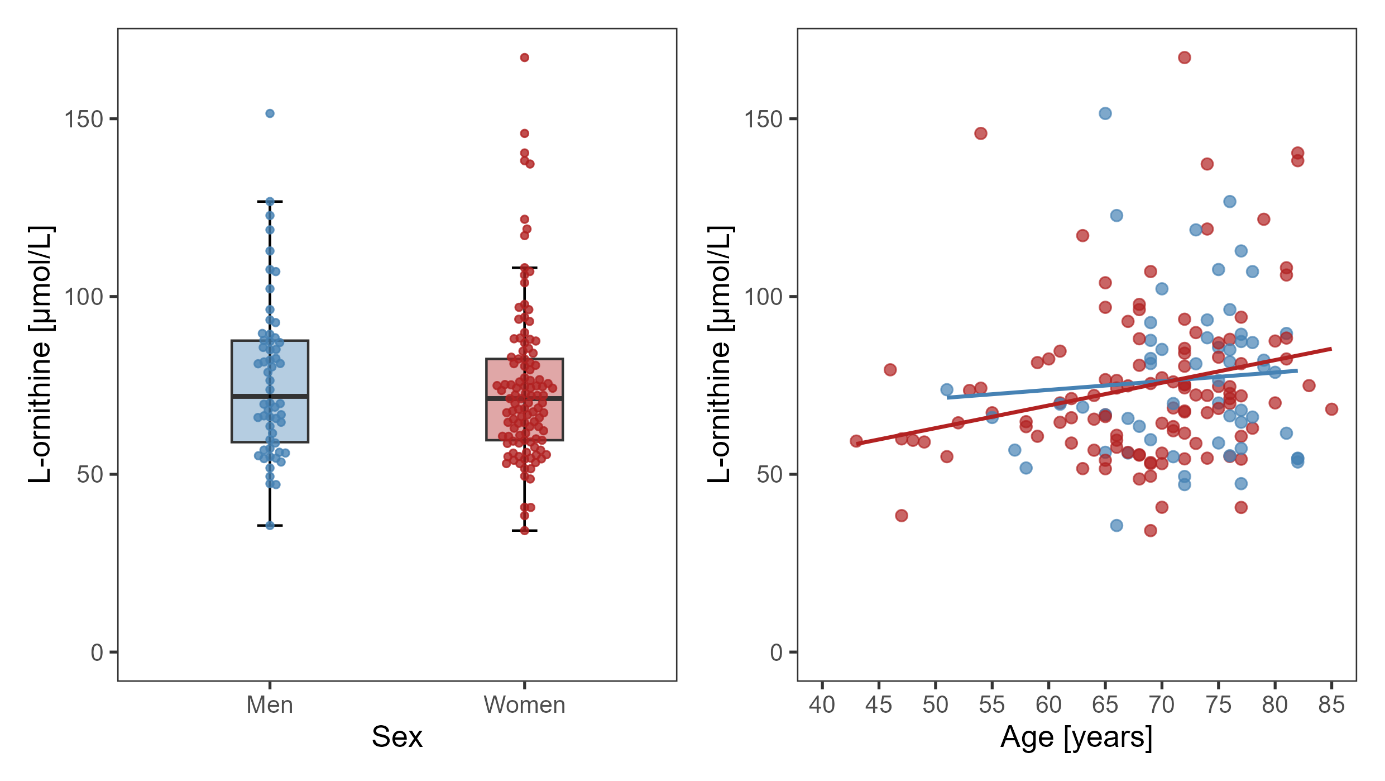
**

**H)
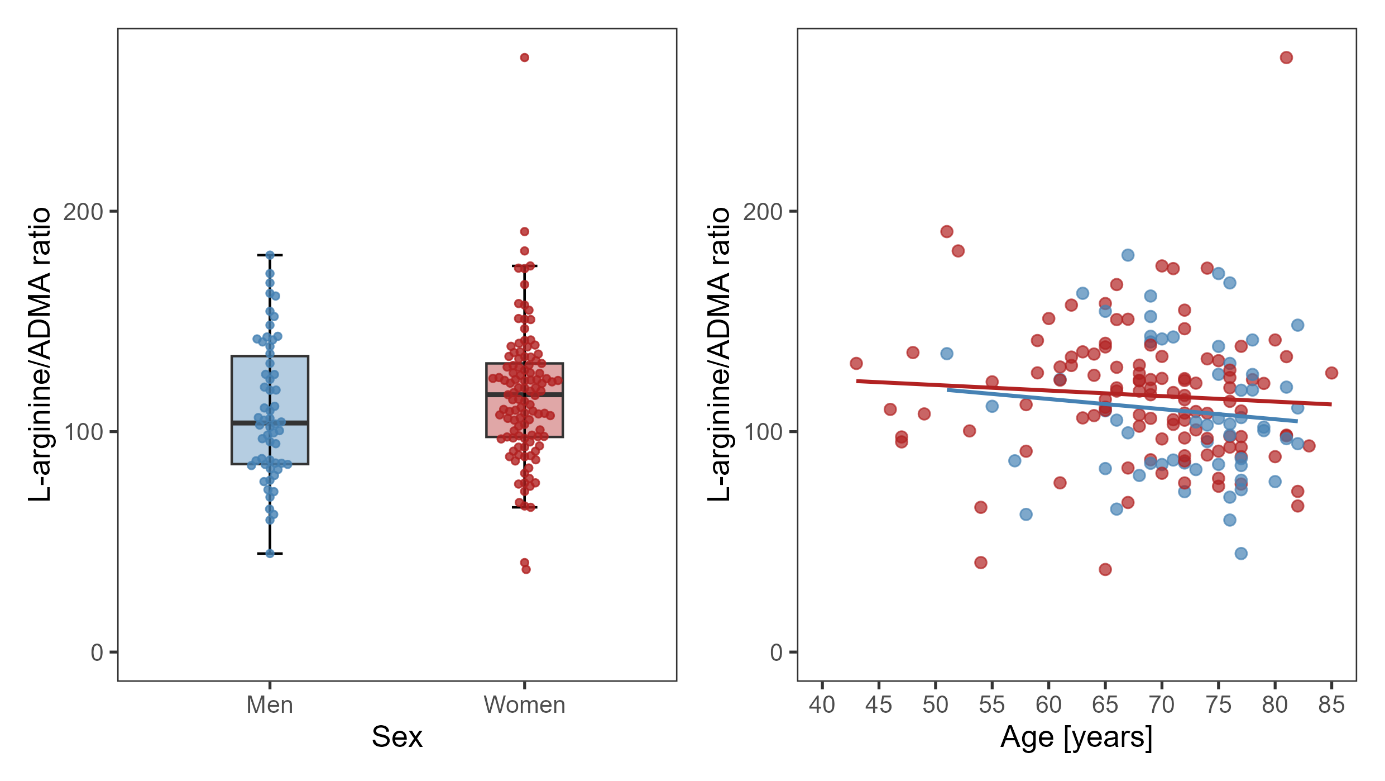
**

**I)
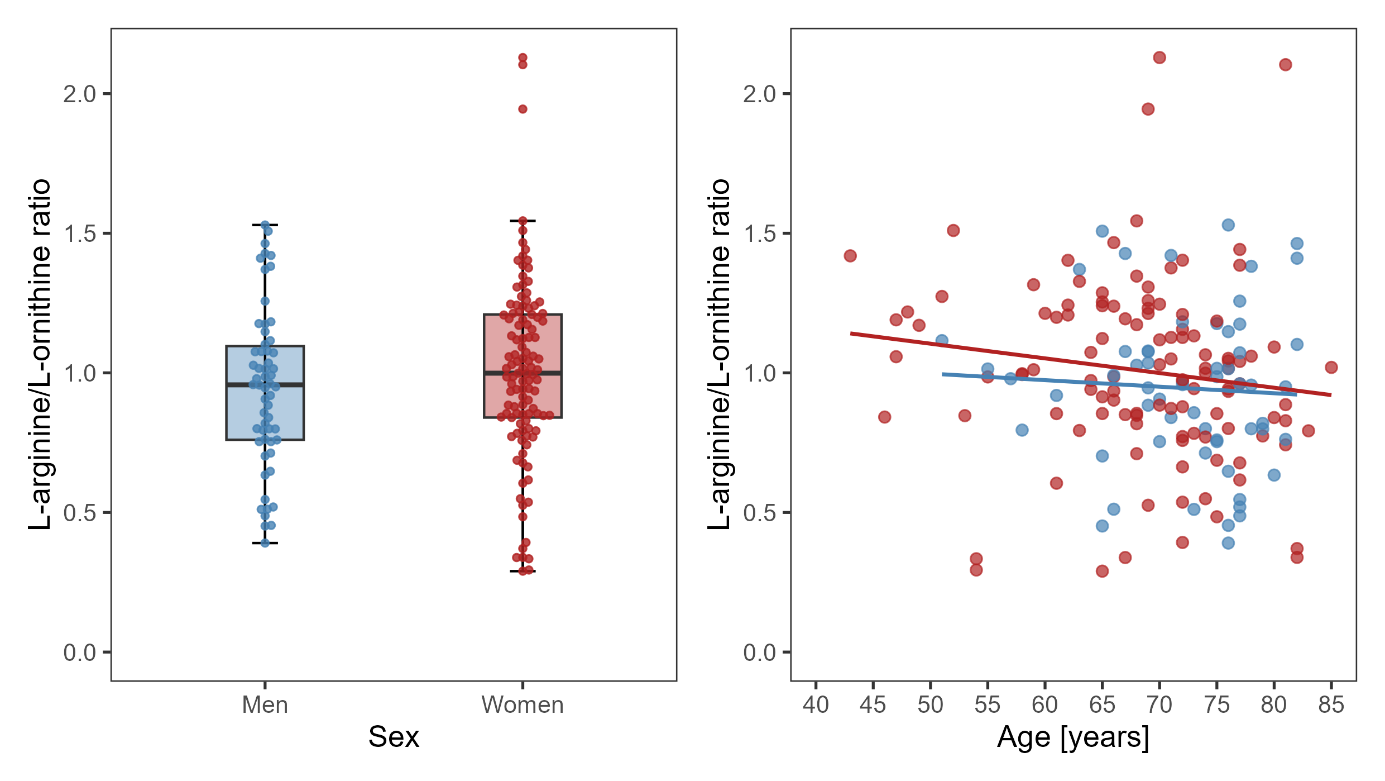
**

**J)
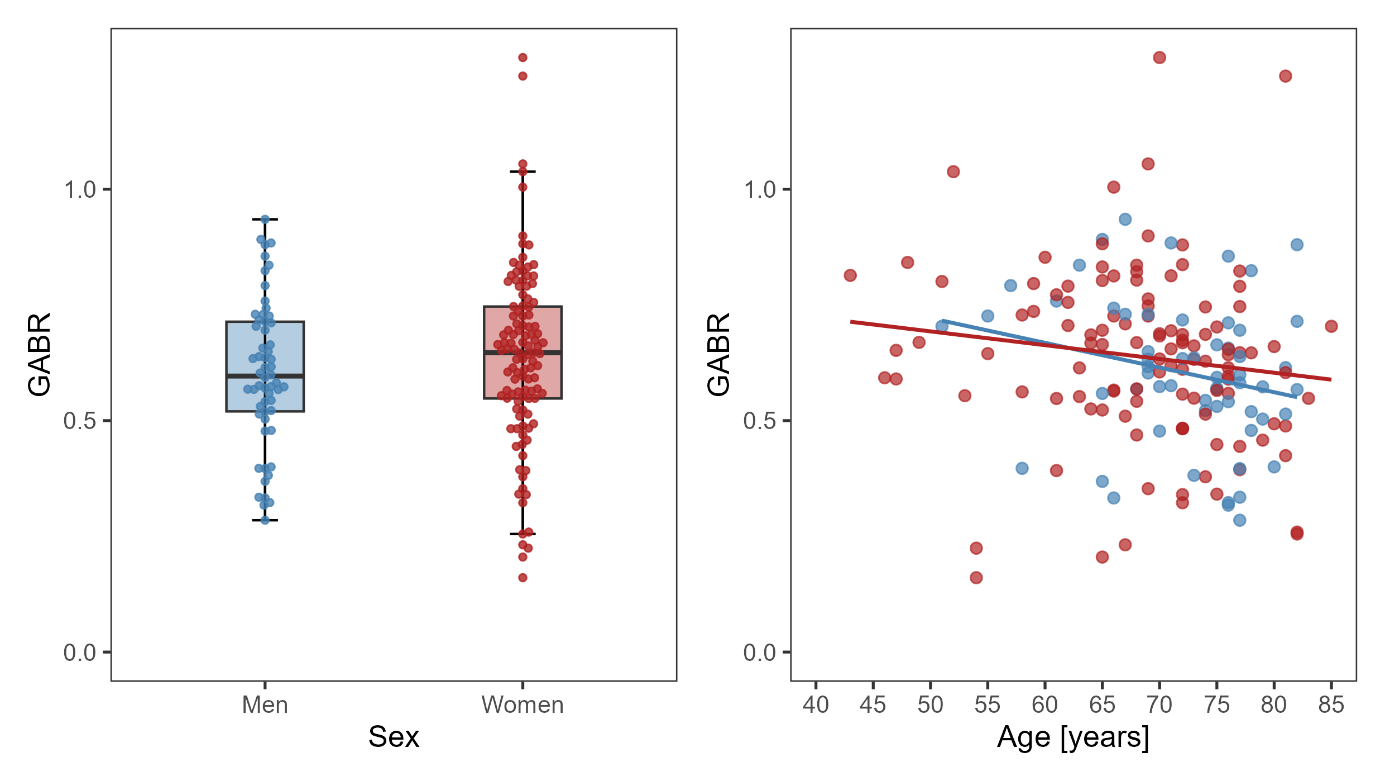
**
